# Functional characterisation of substrate-binding proteins to address nutrient uptake in marine picocyanobacteria

**DOI:** 10.1042/BST20200244

**Published:** 2021-12-09

**Authors:** Benjamin A. Ford, Geraldine J. Sullivan, Lisa Moore, Deepa Varkey, Hannah Zhu, Martin Ostrowski, Bridget C. Mabbutt, Ian T. Paulsen, Bhumika S. Shah

**Affiliations:** 1 Department of Molecular Sciences, Macquarie University, Sydney, Australia; 2 Climate Change Cluster (C3), University of Technology Sydney, Sydney, Australia; 3 ARC Centre of Excellence in Synthetic Biology, Macquarie University, Sydney, Australia

**Keywords:** ABC transport proteins, cyanobacteria, nutrient uptake, *Prochlorococcus*, substrate binding proteins, *Synechococcus*

## Abstract

Marine cyanobacteria are key primary producers, contributing significantly to the microbial food web and biogeochemical cycles by releasing and importing many essential nutrients cycled through the environment. A subgroup of these, the picocyanobacteria (*Synechococcus* and *Prochlorococcus*), have colonised almost all marine ecosystems, covering a range of distinct light and temperature conditions, and nutrient profiles. The intra-clade diversities displayed by this monophyletic branch of cyanobacteria is indicative of their success across a broad range of environments. Part of this diversity is due to nutrient acquisition mechanisms, such as the use of high-affinity ATP-binding cassette (ABC) transporters to competitively acquire nutrients, particularly in oligotrophic (nutrient scarce) marine environments. The specificity of nutrient uptake in ABC transporters is primarily determined by the peripheral substrate-binding protein (SBP), a receptor protein that mediates ligand recognition and initiates translocation into the cell. The recent availability of large numbers of sequenced picocyanobacterial genomes indicates both *Synechococcus* and *Prochlorococcus* apportion >50% of their transport capacity to ABC transport systems. However, the low degree of sequence homology among the SBP family limits the reliability of functional assignments using sequence annotation and prediction tools. This review highlights the use of known SBP structural representatives for the uptake of key nutrient classes by cyanobacteria to compare with predicted SBP functionalities within sequenced marine picocyanobacteria genomes. This review shows the broad range of conserved biochemical functions of picocyanobacteria and the range of novel and hypothetical ABC transport systems that require further functional characterisation.

## Introduction

The marine picocyanobacteria (*Prochlorococcus* [1] and *Synechococcus* [2]*)* are the most abundant photosynthetic organisms in global oceans, playing a critical role in the planetary carbon cycle [3–5]. For growth and photosynthesis, picocyanobacteria must obtain a variety of elements in differing concentrations from the surrounding seawater [6]. This poses a significant challenge for these unicellular organisms in the open ocean, where the availability and speciation of macronutrients (e.g. nitrogen and phosphorus) and micronutrients (e.g. iron and zinc) can be highly variable [6].

The widespread distribution of picocyanobacteria is commonly attributed to the partitioning of ecotypes into distinct ecological niches [7,8]. Genetic adaptation to the nutrient shortage is postulated to be a critical process governing the diversification of both genera [8–16]. With up to 60% of the transport capacity within the already-streamlined picocyanobacterial genomes dedicated to nutrient acquisition systems such as the ATP-binding cassette (ABC) transporters [17–20]. While this mini-review focusses on picocyanobacterial ABC transporters, other predicted transporters (e.g. permeases, ion channels, tripartite ATP-independent periplasmic transporters) may contribute to the uptake of other nutrients [9,11,21,22]. Studies have sought to assess whether changes in gene content among major lineages can provide insight into the global patterns of picocyanobacterial resource use [10,23–25]. Such knowledge is critical to address how climate change-driven changes to nutrient supply (e.g. enhanced stratification of upper ocean layers) may impact future distribution and productivity of picocyanobacterial populations [4,5,26–29].

Notably, omics-based platforms offer an unparalleled capacity to identify patterns of nutrient adaptation [22,25,30,31], including alternative metabolic strategies (mixotrophy), in picocyanobacteria [25,32,33]. Such approaches fundamentally depend on precise functional annotations of cellular nutrient acquisition systems. This is an important caveat as for some nutrient uptake protein families such as the substrate-binding protein (SBP) superfamily (an essential functional element of ABC transporters), evolution has diversified the SBP ligand binding cleft to recognise a myriad of substrates [34]. Consequently, SBPs can often share little (or no) sequence homology [35], limiting the reliability of phylogeny to predict function. Therefore, annotations of SBP function based on remote (<20%) sequence homology [36] limits confidence in predicted nutrient uptake function, resulting in discrepancies between their observed and predicted ligand chemistries [37–39].

Importantly, however, SBPs are structurally and mechanistically conserved with respect to their ligand preferences [36,40]. Modulation of the binding site chemistry, such as through amino acid substitutions, allows the affinity and specificity of SBPs to be tuned to particular ligands without drastically changing the overall architecture of the tertiary fold [41], exemplified in the promiscuity of function in extant [42] and ancestral SBPs [34]. Accordingly, functional differences may not be readily apparent based on phylogenetic relationships. Alternative approaches for classifying SBPs, such as structural comparisons of available SBP structures in the protein data bank (PDB) [36,40], can be extended to predict substrate specificity and molecular evolution of uncharacterised SBPs [34]. Similarly, the increasingly widespread use of deep learning-based protein structure prediction tools (e.g. AlphaFold [43] and RosettaFold [44]) represent an alternative way to test functional predictions [45]. In both cases, these approaches likely require further verification by functional or physiological studies.

This mini-review will focus on relating structurally characterised SBPs in cyanobacteria to predicted SBP annotations in the Cyanorak database, a repository of 97 picocyanobacterial genome sequences [46]. The review highlights the SBP functional space underpinning genomic annotations in marine picocyanobacteria and identify those requiring further validation for unambiguous functional assignment, essential to refining our understanding of how nutrient acquisition shapes ecological trajectories of picocyanobacterial communities.

## Genomic survey of SBP componentry in picocyanobacteria

While structural studies remain a powerful tool for unambiguously determining protein function, few representative picocyanobacterial SBPs have structurally characterised orthologues. Text-based searches of the Cyanorak v2.1 database, comprising sequenced genomes of *Prochlorococcus* (*n* = 43) and *Synechococcus/Cyanobium* strains (*n* = 54) [46], identified 1257 SBPs organised into 26 distinct clusters of orthologous genes (CLOGs) and 12 CLOGs with unknown or hypothetical function associated with high-affinity ABC uptake systems [46]. In this review, each will be referred to by their Cyanorak cluster number (e.g. predicted chitobiose-binding protein ChiE, CK_1342).

**Figure 1.**
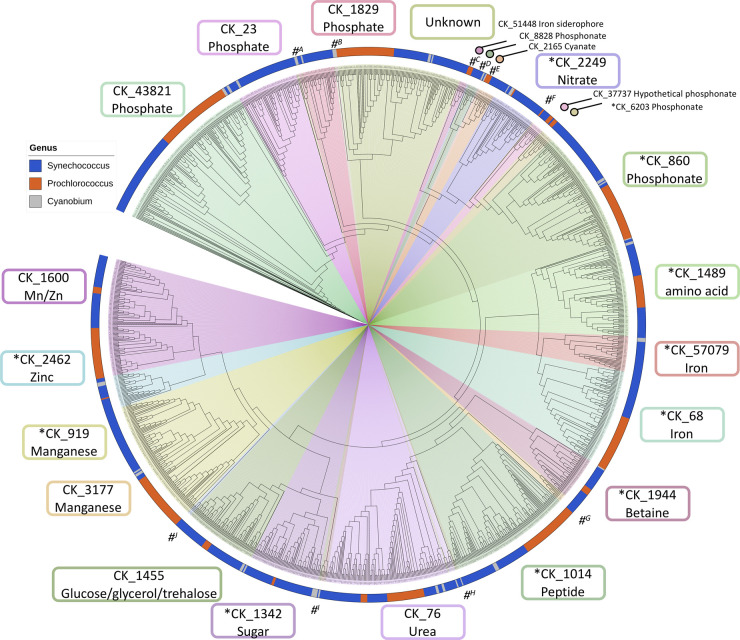
Predicted SBP components from sequenced picocyanobacteria. The cladogram include 1257 SBP sequences extracted from the Cyanorak database [46], drawn using the iToL server [122]. SBP clusters with a structural relative are indicated (*). Clusters with <10 SBP representatives are grouped and marked (#). The cluster numbers for each is as follows: (**A**) CK_57276; (**B**) CK_46636; (**C**) CK_37737; (**D**) CK_4819: (**E**) CK_34148; (**F**) CK_33184; (**G**) CK_3177; (**H**) CK_31409; (**I**) CK_3072, 23352; (**J**) CK_40935 The protein alignment using MAFFT (v7.453) [123], with the global pair alignment and phylip output options, was used to construct the cladogram using FastTree 2.1 [124]. Colours representing the three genera: *Synechococcus* (blue outer circle), *Prochlorococcus* (orange outer circle) and *Cyanobium* (grey outer circle) are included to show the phylogenetic partitioning of genes within SBP clusters.

Highly abundant picocyanobacterial SBPs (Figure 1) include those of unknown function (e.g. CK_264) and those predicted to mediate uptake of organic carbon (CK_1342, 1455). Other abundant SBPs, such as for the uptake of urea (CK_76) and phosphate (CK_43821) have been characterised using physiological assessments including growth- and uptake assays, and proteomic approaches [30,31,47]. In addition, other less prevalent SBP clusters currently have no known or predicted function.

**Figure 2.**
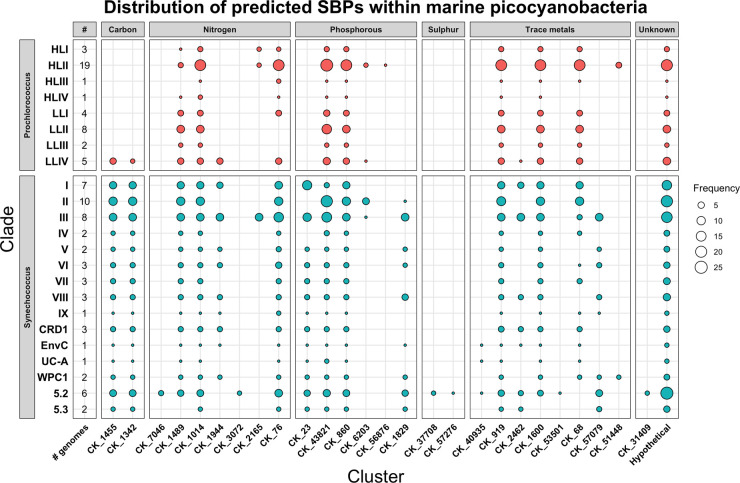
Patterns in distribution of predicted SBPs within marine picocyanobacterial subclusters and clades. Each column corresponds to a particular SBP cluster of orthologous genes contained within the Cyanorak database. Cluster numbers are provided except for those of unknown function which are grouped together. Clusters, identified by their Cyanorak numbers, are grouped according to their major nutrient class (i.e. carbon, nitrogen, phosphorus, sulfur, metals, and unknown). Their presence for individual clades of picocyanobacteria (*Synechococcus, Prochlorococcus* and *Cyanobium* in subcluster 5.2) is indicated, highlighting clade-level patterns in their distributions. The size of the circle corresponds to the frequency of predicted SBPs in the clades per cluster. The clusters comprising the hypothetical/unknown group are: CK_264, CK_2249, CK_3177, CK_4819, CK_4821, CK_8828, CK_23352, CK_31409, CK_32631, CK33184, CK_34148, CK_37737, CK_44411, CK_44797, CK_46634, and CK_46636. A dataset containing each SBP identified per sequenced genome is provided as Supplementary Data S1.

As predicted picocyanobacterial SBPs are not uniformly distributed across all clades, the presence of specific clusters in distinct lineages could provide insight into environmental niche preferences (Figure 2). Some picocyanobacterial representatives from subcluster 5.2, including *Synechococcus* and *Cyanobium* isolates, possess all predicted SBP clusters in their genomes. Given most subcluster 5.2 isolates are found in coastal or estuarine waters [48], the high-nutrient environment likely led to less extensive genome streamlining [9,49,50] and thus a greater diversity of nutrient acquisition systems being retained [51]. The potential influence of the environment on phyletic distributions of SBP clusters in other picocyanobacterial clades highlights fundamental differences in their retained genetic capacity for high-affinity nutrient uptake, likely reflecting the adaptation of these taxa to specific environmental conditions. For example, every sequenced picocyanobacterial strain retains at least one predicted SBP gene for the uptake of growth-limiting nutrients like phosphorus (e.g. CK_860), and trace metals like iron (e.g. CK_68), while the functional capacity for the predicted uptake of organic carbon (CK_1342, CK_1455) or osmoprotectants (CK_1944) occur in select strains, likely correlating with distinct environments [25,31,51–53].

Notably, almost all strains of picocyanobacteria harbour genetic capacity, based on genomic annotations, to uptake organic nutrients (e.g. organic phosphonates or sugars). Given marine picocyanobacteria predominate in oligotrophic regions of the world's oceans [4], a mixotrophic approach, where inorganic nutrient uptake is supplemented by scavenging organic nutrients via high-affinity ABC transporters, could impart a distinct evolutionary advantage to acquire essential, often limiting nutrients [11,32,54]. This more nuanced metabolic strategy has been hinted at since the publication of early genomes [18–21] and may directly explain the prevalence of ABC transporters in picocyanobacterial genomes [25].

## SBP structures from cyanobacteria across core nutrient classes

The quantitative relationships between intra- and extracellular nutrient pools underpins the concept of ecological stoichiometry, driving ocean biogeochemistry [6]. While six essential elements (C, H, N, O, P, and S) comprise most of the organic matter (e.g. macromolecules, genetic material, biological membranes), all organisms require a suite of additional inorganic ions (e.g. trace metals) to ensure the proper functioning of biological machinery [6,8,55]. Ecological stoichiometry, exemplified by the Redfield ratio, links the availability of biogeochemical carbon, nitrogen, phosphorus and iron in the oceans [56], and underpins ecosystem models at the individual, population, community and global scales [57]. The specific uptake of key nutrient classes by picocyanobacteria via ABC transporters [17–20] relies on the SBP subunit, whose function can be determined by characterising individual protein structures and their associated atomic features. A summary of structurally characterised SBPs from cyanobacteria, is presented in Table 1.

**Table 1:** Known SBP structures from cyanobacteria^a^

Group	Substrate	Protein	Organism(s)	PDB codes	Structural Cluster^b^	Ref.	Putative gene orthologues in Cyanorak^c^
Carbon	Bicarbonate	CmpA	*Synechocystis* sp*.* PCC 6803	2I48, 2I49, 2I4B, 2I4C	F-I	[68]	CK_00009155
	Predicted chitobiose	MITs9220_121, ChiE	*Synechococcus* sp*.* MITS9220	6WPM, 6WPN	D-II	our unpublished data	CK_00001342
Nitrogen	Nitrate/nitrite	NrtA	*Synechocystis* sp*.* PCC6803	2G29	F-I	[83]	CK_00003072/00002249
	Amino acids/amide	Ava_0465	*Anabaena variabilis* ATCC 29413	4NQR, 4NV3, 4OAT, 4OG2, 4OTZ, 4QYN, 4RDC	B-II	Unpublished	CK_00001489/00001014
	Glutamate	Slr1257	*Synechocystis* sp*.* PCC 6803	1II5, 1IIT, 1IIW	F-IV	[125]	
	Glutamate	N/A	*Nostoc punctiforme* PCC 73102	2PYY	F-IV	[126]	
Phosphorous	Phosphite/ Phosphonate	PhnD1	*Prochlorococcus marinus* sp. MIT 9301	5LQ5, 5LQ8	F	[97,103]	CK_00000860/00006203/00056876
	Phosphite	PhnD2	*Prochlorococcus marinus* sp. MIT 9301	5LV1	F	[97,103]	CK_00000860/00006203/00056876
	Phosphite/ Phosphonate	Tery_0366, PtxB	*Trichodesmium erythraeum* IMS101	5JVB, 5LQ1	F	[97]	
Sulfur			N/A				
Metal	Zinc	ZnuA (or ZntC)	*Synechocystis* sp. PCC 6803	1PQ4, 2OV1, 2OV3	A-I	[113,114]	CK_00002462CK_00057079/00000068
	Iron, Fe(III)	Tery_3377	*Trichodesmium erythraeum* IMS101	6G7N, 6G7P, 6G7Q	D-IV	[69]	CK_00057079/00000068
	Iron, Fe(II)	FutA1	*Synechocystis* sp. PCC 6803	2PT1, 2PT2, 3F11	D-IV	[104]	CK_00000919
	Iron, Fe(III)/Fe(II)	FutA2	*Synechocystis* sp. PCC 6803	2VOZ, 2VP1	D-IV	[105]	CK_00002462
	Manganese	MntC	*Synechocystis* sp. PCC 6803	1XVL, 3UJP, 4IRM	A-I	[115]	CK_00057079/00000068

a This table uses the same major nutrient classes as Figure 2 (i.e. C, N, P, S, metals, and other). SBPs for the uptake of particular substrates are shown, grouped by organism, along with their corresponding PDB identifiers and Cyanorak orthologues, if known;

b Designated based on structural classifications of the Poolman group [36,40];

c Defined by Cyanorak cluster identifier [46].

### SBPs for carbon uptake

As photoautotrophs, picocyanobacteria are typified by their ability to use photosynthesis to convert inorganic carbon (C*_i_*) into biologically useful (labile) forms of organic carbon that are then cycled through the environment [6,58]. Marine environments act as vast reservoirs for C*_i_*, which ultimately originates from atmospheric carbon dioxide and dissolved carbonate minerals [59–61]. Carbon concentrations are generally highest in the photic zone due to the concentration of primary production in this region of the oceans [62]. Picocyanobacteria have been predicted to access organic carbon [25,33,63,64], with studies demonstrating the uptake of glucose (a molecule containing no growth-limiting elements) in marine picocyanobacteria [65,66], and light-mediated mixotrophy as a strategy to overcome inorganic nutrient limitation [67].

**Figure 3.**
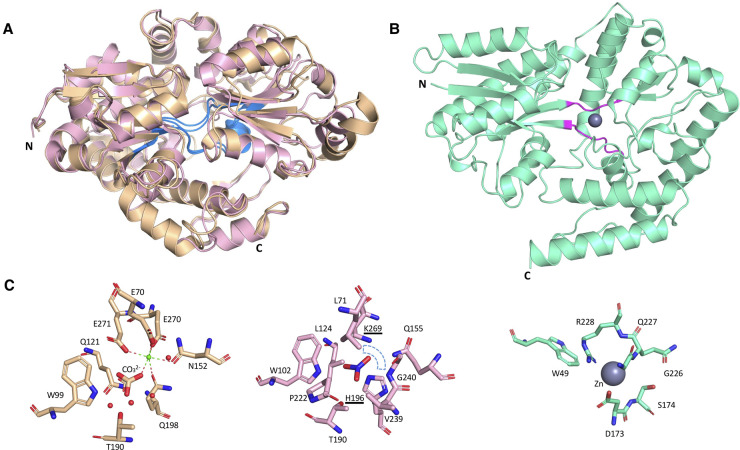
Structures of CmpA, NrtA and annotated ChiE. (**A**) The overlaid cartoon representation of two related protein structures, CmpA (PDB ID: 2I48, wheat) and NrtA (PDB ID: 2G29, pink) (r.m.s.d = 0.812 Å),) showing the defining hinge architecture of Cluster F (blue). (**B**) The cartoon representation of the annotated chitobiose-binding protein 3D structure (ChiE, CK_1342) from *Synechococcus* MITS9220 (PDB ID: 6WPM, green) showing defining hinge architecture of Cluster D (pink) and zinc in the binding cleft. (**C**) The ligand binding cleft for Ca^2+^-mediated CmpA (wheat), NrtA (pink) with key substituted residues (underlined) and region of positive charge (blue dashed sector), and annotated ChiE (green) with zinc co-ordination site within the binding cleft.

The structure of only one cyanobacterial carbon-binding protein, CmpA, from the freshwater *Synechocystis sp.* PCC 6803 has been published to date [68]. This protein binds C*_i_* (CO_3_^2^ and HCO_3_^−^) at the interdomain cleft (Figure 3). Binding occurs in a pH-dependent manner, with CmpA forming part of an operon induced under low CO_2_ conditions [68]. For HCO_3_^-^_,_ binding occurs in an anionic cage, dependent on the presence of calcium (Ca^2+^) to balance the negative charge on the carbonate ion, reminiscent of concomitant metal-anion binding observed for other cyanobacterial binding proteins [69] and appears physiologically relevant. This is further supported by studies of allosteric regulation in related bicarbonate-binding proteins [70] and transcriptomic analysis [71].

As part of the carbon-concentrating machinery (CCM) in freshwater *Synechocystis sp.* PCC 6803 [68], CmpA assimilates dissolved C*_i_*. This elevates CO_2_ concentrations around RuBisCo within the carboxysome, facilitating CO_2_ fixation under low CO_2_ conditions [11,72]. In picocyanobacteria, CmpA (CK_9155) orthologs are only found in two sub-cluster 5.2 strains, *Synechococcus* WH5701 and *Cyanobium* PCC6307, suggesting CmpA-based CCM is not a prevalent function for marine picocyanobacteria. Given higher concentrations of carbonate within marine environments [73] and the divergent origin of marine picocyanobacterial CCM compared with other cyanobacteria [11], C*_i_* uptake using high-affinity ABC transport machinery may not be necessary for picocyanobacteria in the marine context [11].

In contrast, many marine picocyanobacteria possess conserved SBPs predicted to uptake organic carbon (particularly sugars). These include CK_1342 — a cluster conserved across all *Synechococcus* strains yet largely absent from *Prochlorococcus*, and annotated to bind chitobiose (ChiE), CK_1455 (annotated to uptake glucosylglycerol/sucrose/trehalose) — a cluster conserved across all *Synechococcus* and *Prochlorococcus* LLIV strains, and a hypothetical sugar-binding protein cluster, CK_34148 present in a single *Synechococcus* clade IIIb strain (A15-28). Given the limited reliability of these gene annotations, these predicted functions may indeed be spurious and so a cautious interpretation of these annotations is warranted.

Our group recently solved two crystal structures (PDB ID: 6WPM, 6WPN) for a CK_1342 protein from an open ocean *Synechococcus* strain MITS9220, showing this protein conforms to other structural Cluster D proteins (Table 1) that interact with carbohydrates [36,40]. Ongoing ligand binding tests suggests zinc may be bound within the binding cleft (PDB ID: 6WPM) of one of the CK_1342 protein. We hypothesise that zinc may play a role in concomitant ligand binding of CK_1342 protein as seen for CmpA — a notion further reinforced by changes in the 3D structural elements between the zinc-free (PDB ID: 6WPN) and zinc-bound (PDB ID: 6WPM) forms. Uncovering evidence of SBPs involved in organic carbon uptake remains an important step in exploring the presence of mixotrophy in picocyanobacteria.

### SBPs for nitrogen uptake

Nitrogen (N) is critical for protein and nucleic acid synthesis in picocyanobacteria and broadly regulates phytoplankton primary production [74]. Acquisition of inorganic N by most marine picocyanobacteria is performed by secondary membrane-bound transporters, such as nitrate permeases (NrtP or NapA) [18,75,76], or ammonium permease (Amt1) [77]. This contrasts with the active transport favoured by freshwater cyanobacteria, which utilise a high-affinity ABC transporter, NrtABCD [78–81]. The SBP component (NrtA) binds both nitrate (NO_3_^−^) and nitrite (NO_2_^−^) with comparable affinities (*K_D_* = 0.32 µM and 0.34 µM, respectively) [82]. Despite facilitating N uptake, NrtA is more closely related in structure to CmpA of the bicarbonate uptake system (see Carbon section) [81,83] than to SBPs for other nitrogenous compounds, with both proteins belonging to a cluster (F-I) that specifically bind trigonal planar oxyanions [36,40].

Unlike CmpA (PDB ID: 2I48) where HCO_3_^−^ -binding requires Ca^2+^, in the case of NrtA (PDB ID: 2G29) the negative charge of NO_3_^−^ is balanced by substitution of basic residues (K269 and H196) to alter binding cleft chemistry (Figure 3). Comparing the binding clefts of CmpA and NrtA indicates anion selectivity is governed mainly by modulating charge such that binding occurs either via a co-ordinated metal ion (CmpA) or directly to charged sidechains (NrtA), indicating a level of sophistication in substrate preference that limits promiscuity in function.

As with organic carbon, picocyanobacteria are predicted to uptake organic N compounds, including amino acids, peptides, and quaternary amines [25,33,46]. Known structurally characterised SBPs from freshwater *Synechocystis*, include a glutamate-binding protein (PDB ID: 1II5, 1IIT, 1IIW) and an amino acid/amide-binding protein from the filamentous *Anabaena* (PDB ID: 4NQR, 4NV3, 4OAT, 4OG2, 4OTZ, 4QYN, 4RDC). These SBPs are homologous to annotated SBP componentry for these nutrient sources in picocyanobacteria (e.g. clusters CK_1489, CK_7046, and CK_1944, respectively).

Similarly, *A. variabilis* possesses what appears to be a highly promiscuous amino acid-binding protein (PDB ID: 3I6V), based on the function assigned in the PDB structure deposition. Orthologues of this amino-acid binding protein in marine picocyanobacteria occur in cluster CK_1489, an SBP predicted to bind acidic or polar amino acids. This SBP is conserved across all *Synechococcus* clades, except for a subcluster 5.2 strain (*Cyanobium* CB101) and all subcluster 5.3 strains. The phyletic distribution of this SBP in *Prochlorococcus* is mainly limited to low-light (LL) *Prochlorococcus* strains. Only one LL strain (MIT0601) lacks the SBP gene from this cluster, whereas the gene is almost entirely absent from high-light (HL) strains. Such a marked distribution indicates the cognate ligand for this cluster is likely present across different environments, but, stratified down the water column.

All sequenced picocyanobacteria, except *Prochlorococcus* LLII/III strains possess unvalidated SBP componentry annotated to uptake urea (UrtA, CK_76). Experimental evidence indicates picocyanobacteria display higher growth rates in the presence of urea [84], with some strains using urea as a sole nitrogen source [85] — further reinforced by characterisation of urease genes in *Prochlorococcus* PCC9511 [86] and *Synechococcus* WH7805 [87]. Some strains (e.g. from *Synechococcus* Clade IIIa) harbour ‘orphan’ urtA genes (that is, without additional ABC transporter subunits) close to phage-associated genes, indicating these SBPs may be propagated within the environment through lateral gene transfer events and possibly interact with alternative ABC transport machinery to facilitate urea uptake in these strains. The unresolved biological role and interacting partners for these orphan SBPs requires additional physiological and functional studies.

Like urea, additional organic nitrogen uptake may also occur through predicted cyanate transporters (CK_2165). The distribution of these transporters appears clade-specific in *Synechococcus* (Clade III) [22], however, these are yet to be functionally validated. As nitrogen is a major limiting nutrient in marine environments, mixotrophic strategies for the acquisition of organic nitrogen may be crucial for picocyanobacteria [33,63,88–90]. However, the identity of organic nitrogen species accessible to individual strains remains an open question. Additional structural or biochemical validation of hypothetical proteins, such as the conserved predicted urea uptake gene (CK_76), the ambiguous peptide or nickel transporter (CK_1014), and clade-specific cyanate transporter (CK_2165) would assist with confidently assigning functions to these SBPs and their associated metabolic pathways.

### SBPs for phosphorus uptake

Phosphorus (P) is another important limiting nutrient across marine environments, serving as a principal element in cellular macromolecules and energy stores (e.g. DNA, RNA and ATP) [91–96]. Picocyanobacteria possess multiple phosphorus acquisition pathways for both inorganic (phosphate and phosphite) and organic (phosphonate) P compounds [93,94,97], reflecting their need for this essential nutrient.

Marine picocyanobacteria use the high-affinity Pst transport system to uptake phosphate [98]. The SBP in this system, PstS, is highly conserved in picocyanobacteria [11,99], with some strains possessing multiple copies of the Pst*S* (clusters CK_23, CK_43821). Differences in Pst*S* copies between picocyanobacterial strains likely reflects adaptation to phosphorus availability. For example, *Synechococcus* WH8102, which originate from P-deplete environments [100] possesses two copies of PstS (CK_43821) and one copy of the additional PstS2 (CK_23) [11], while strains in P-replete waters (e.g. *Synechococcus* CC9311) have only a single copy of PstS2.

Quantitative PCR and radiolabelled uptake studies suggest freshwater *Synechocystis* use PstS proteins to sense changes in external inorganic phosphate concentrations [99]. Similarly, the cyanobacterial-specific SphX protein is also known to be up-regulated under phosphorus stress in *Synechococcus* WH8102 [31], and acts as a functional homologue in PstS-deficient freshwater strains [101]. Despite their possible role in general cellular stress responses [31,52,102], characterisation of PstS and SphX has only encompassed transcriptomic or proteomic responses to phosphorus stress [31,52,102], providing further scope to characterise these SBPs structurally.

**Figure 4.**
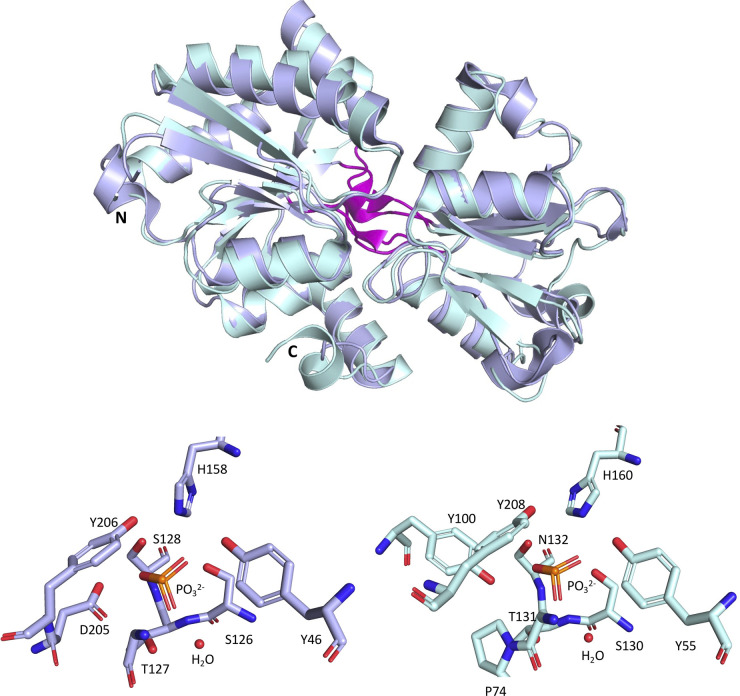
Structures of *Prochlorococcus* MIT9301 PhnD1 and PhnD2 in complex with phosphite. Above, the overlaid structures of PhnD1 (PDB ID: 5LQ5, lilac) and PhnD2 (PDB ID: 5LV1, blue) (r.m.s.d = 1.05 Å) showing the defining hinge architecture of Cluster D (magenta). Below, the key ligand-binding residues for each of these proteins are shown (left) for PhnD1 and (right) for PhnD2.

All picocyanobacterial strains also possess the predicted phosphonate-binding protein PhnD1 (cluster CK_860), while some *Prochlorococcus* HLII/LLIV and *Synechococcus* clade II isolates also have a second, divergent copy, PhnD2 (CK_6203) [94]. A single *Prochlorococcus* HLII strain (MIT9314) even possesses a third copy (PhnD3, CK_56876), hypothesised to correspond to niche adaptation in low-phosphorus environments [46]. *Prochlorococcus* MIT9301 PhnD1 and PhnD2 proteins have been structurally characterised (PDB ID: 5LQ5/5LQ8 and 5LV1, respectively) (Figure 4) [94,97], with PhnD1 shown to bind phosphite (PO_3_^2−^) with nanomolar affinities (K_D_ = 50–120 nM) [94,97], and also recognise inorganic phosphate (K*_D_* = 55–200 μM) and methylphosphonate (K*_D_* = 40–110 μM), though with binding constants comparatively weaker than PO_3_^2−^ [94,97]. Conversely, *Prochlorococcus* MIT9301 PhnD2 displays nanomolar affinities for organic methylphosphonate (K*_D_* = 80 nM) and low-micromolar affinity for PO_3_^2−^ (K*_D_* = 2 μM) [94], but no measurable affinity for any other phosphorus source.

Between *Prochlorococcus* MIT9301 PhnD1 and PhnD2, the biggest change in the binding cleft (Figure 4) appears to be substitution of an asparagine residue in a conserved -serine-threonine-serine- motif (PhnD1, S126-T127-S128) to form -serine-threonine-asparagine- (PhnD2, S130–T131–N132), respectively. The substitution of asparagine favours interactions with hypophosphite (H_2_PO_2_^−^) in related proteins, however, PhnD2 in *Prochlorococcus* displays no measurable affinity with hypophosphite [97]. All other key ligand-binding residues are contained at structurally conserved sites. Engagement of the carboxyl group of an aspartic acid (PhnD1, D205) found at the beginning of β-strand_10_ has been shown to tune PhnD1 in *Prochlorococcus* to recognise phosphite [97]. This residue is absent from PhnD2, resulting in a weaker affinity for phosphite. While interactions from semi-conserved tyrosine (π-donor) with phosphite (P-H acceptor) lead to the formation of P-H…π bonds that stabilise phosphite in highly specialised relatives (PtxB from *Trichodesmium;* PDB ID: 5JVB/5LQ1) [97], modulation of hydrophobic residues in the binding cleft has been shown to provide steric selectivity for hypophosphite in the related HtxB [97].

Despite broad structural conservation of PhnD proteins and their relatives (PtxB, HtxB), the precise molecular determinants of ligand binding, as discussed above, are due to the embellishments of the conserved fold around the binding cavity [97] and the protonation state of the ligand [103]. As with N, identifying the specific P-sources that can be utilised by different picocyanobacterial strains, via their multiple P acquisition pathways, would be a useful avenue of exploration to understand niche differentiation across the marine environment.

### SBPs for trace metal uptake

#### Iron

Iron (Fe) is an essential micronutrient for optimal photosynthetic functioning in picocyanobacteria, and under certain circumstances can be growth-limiting [6,9,11]. Both marine (e.g. picocyanobacteria and the filamentous *Trichodesmium erythraeum)* and freshwater cyanobacteria (e.g. *Synechocystis* PCC 6803) use similar SBPs for Fe uptake such as the iron-deficiency induced protein A, IdiA (also referred as FutA) [69].

**Figure 5.**
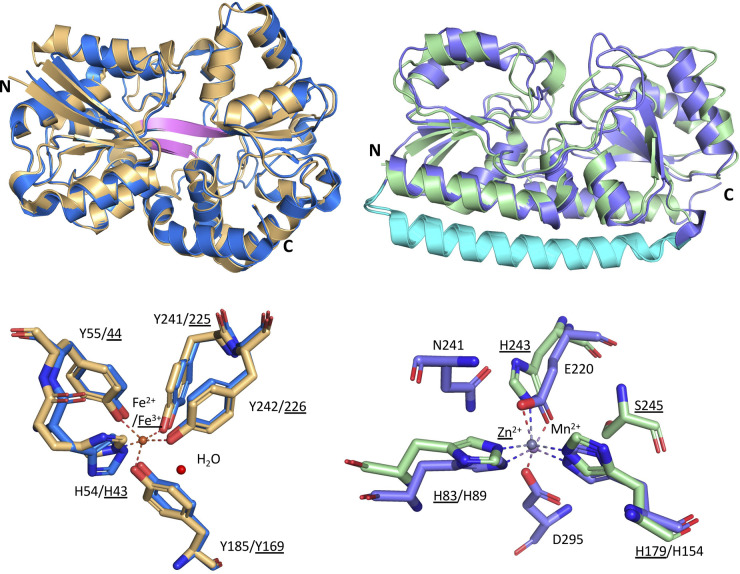
Structures of metal-binding proteins from *Synechocystis* PCC 6803. Above (left), the overlaid structures of FutA1 (PDB ID: 3F11, yellow) and FutA2 (PDB ID: 2VP1, blue) from *Synechocystis* PCC 6803 (r.m.s.d. = Å) with defining hinge architecture coloured pink. Above (right) overlaid structures of ZnuA (PDB ID: 1PQ4, green) and MntC (PDB ID: 1XVL, purple) from *Synechocystis* PCC6803 (r.m.s.d = 1.03 Å) showing the defining hinge architecture of Cluster A (turquoise). Below, the key ligand-binding residues for each of these proteins are shown (left) for FutA1 and FutA2 and (right) for ZnuA and MntC (overlaid).

Two distinct iron-binding proteins (FutA1, FutA2) from *Synechocystis* PCC 6803 have been structurally characterised. These bind iron in two alternative oxidation states: FutA1 (PDB ID: 2PT1, 2PT2, 3F11) binds ferrous iron (Fe^2+^) [104], while FutA2 (PDB ID: 2VOZ, 2VP1) binds ferric iron (Fe^3+^) (Figure 5) [105]. Biochemical evidence indicates FutA1 does not behave as a classical SBP for nutrient uptake, but instead interacts intracellularly with photosystem II to alleviate photoinhibition during iron deficiency [106,107]. In contrast, FutA2 is exported to the periplasm via the twin-arginine (Tat) pathway [105], and likely functions as a typical periplasmic SBP for iron uptake. In *Synechocystis* PCC 6803, both FutA1 and FutA2 utilise a conserved tyrosine clamp motif to bind Fe. This consists of four tyrosine and one histidine residues which coordinate either ferrous or ferric iron [104,105] (Figure 5), raising the question of how these proteins balance the different oxidation states of Fe. The orthologous FutA1 protein from marine *T. erythraeum* has also been structurally characterised (PDB ID: 6G7N, 6G7P, 6G7Q) [69], however, the mechanisms by which these two cyanobacteria assimilate Fe appear different. In *T. erythraeum,* Fe binding is mediated by an organic siderophore (Fe-citrate) in contrast with the direct uptake of ionic iron by the FutA1/FutA2 proteins in *Synechocystis* PCC 6803 [108].

While the mechanisms for iron acquisition by marine picocyanobacteria would be expected to mimic *T. erythraeum* more closely, there is evidence that siderophore-mediated uptake of Fe is rare among picocyanobacterial strains [11,109]. We speculate this correlate with an eco-physiological strategy reflective of the abundance of the higher oxidation state Fe^3+^ relative to that of Fe^2+^ (discussed further below). Within the Cyanorak database, three clusters corresponding to Fe-specific SBPs are annotated; IdiA1 (CK_57079), IdiA2 (CK_68) and IdiA3 (CK_57080). IdiA2 is widely distributed among picocyanobacteria, except for *Prochlorococcus* HLIV strains, *Synechococcus* clades V, VIII and subclusters 5.2 and 5.3. In contrast, IdiA1 is only found in *Synechococcus* clades III, VI, IX, and WPC, as well as IdiA2 lacking *Synechococcus* clades V, VIII, and subclusters 5.2 and 5.3. However, IdiA3 is found only in two *Synechococcus* clade VIII strains (RS9909 and RS9917). The presence of complementary predicted iron-binding proteins (IdiA1, IdiA2 and IdiA3) in picocyanobacterial strains reinforces the potential use of multiple iron acquisition strategies.

Unlike their freshwater counterparts, marine picocyanobacteria appear not to have SBPs to mediate the uptake of iron in its lower oxidation state (Fe^2+^), possibly reflecting the fact that the uncomplexed, dissolved Fe^2+^ is rapidly oxidised to Fe^3+^ in surface ocean waters and extremely rare [110]. Instead, dissolved Fe in the oceans primarily exists in complex with organic compounds, including colloids and iron-binding ligands [110]. This may reflect greater competition for ferrous, rather than the less biologically useful ferric form, in marine environments, coupled with a biochemical strategy inherently favouring the reduction in ferric iron. The impact of very low iron concentrations on evolved affinities and specificities for Fe-specific SBPs, alternative pathways for the uptake of reduced Fe [109], and the precise mechanisms of microbial-mediated feedback loops [109], remain highly topical areas for further investigation of trace metal acquisition, particularly iron.

#### Zinc and manganese

Zinc (Zn) and manganese (Mn) are essential micronutrients needed for the maintenance of photosynthetic apparatus, their environmental distribution varies [11,55,111,112], with free Mn concentrations even lower than those of Zn and generally higher at the ocean's surface [55]. SBP componentry for zinc, ZnuA (formerly called ZntC, PDB ID: 1PQ4, 2OV1, 2OV3) and manganese, MntC (PDB: 1XVL, 3UJP, 4IRM) uptake has been demonstrated for *Synechocystis* PCC 6803*,* which conforms to the Zn and Mn-binding archetypes from other bacterial species, such as *E. coli* [113,114].

*Synechocystis* PCC 6803 ZnuA and MntC proteins employ similar coordination for each metal ion, engaging these in a histidine-rich binding pocket [113,115]. Specificity for each metal ion is governed by slightly different sidechains to satisfy each ion's coordination geometry. *Synechocystis* PCC 6803 ZnuA use a triad of histidine residues (Figure 5), with the fourth Zn coordination bond satisfied by an exogenous water. MntC uses two histidine and two acidic sidechains co-ordinate the octahedral Mn ion, with one acidic sidechain contributing two coordination sites [108] (Figure 5). An additional structural embellishment, specific to Zn-binding proteins, is a histidine-rich loop that serves to increase available zinc concentrations close to the binding cleft [108,113,114].

In marine picocyanobacteria, the predicted Zn-binding protein is annotated as ZnuA (CK_2462) [116,117]. This specific cluster (CK_2462) is almost entirely absent from *Prochlorococcus* (except for strain MIT9303), and is found in select members of *Synechococcus* clades I, III, VIII, CRD1 and XX, and sub-clusters 5.2 and 5.3. In contrast, the predicted Mn-binding protein, MntC (CK_919), is found in all picocyanobacteria except one subcluster 5.2 strain (*Cyanobium* CB0205), with some strains (*Synechococcus* TAK9802 and BIOS-E4-1) encoding extra copies. Another SBP (CK_1600) annotated as either a Zn- or Mn-specific SBP is found in all strains except some from subcluster 5.2 and 5.3. The presence of a dedicated Mn-binding protein (CK_919) would indicate the latter (CK_1600) may bind Zn. However, physiological work has shown it is up-regulated under Fe stress [118], and competitively inhibited by cobalt (Co) [119]. The similarities in ligand-binding between Zn and Mn mean that conclusive resolution of substrate specificity will depend upon future structural and biochemical validation.

### Additional trace metals

Picocyanobacterial biomass consists of around 30 naturally occurring elements. In addition to the macro- and micro-nutrients outlined above, picocyanobacteria also likely acquire microelements using a range of both SBP-mediated and other transport mechanisms [6]. For example, copper (Cu) uptake is mediated by a P_1_-type ATPase rather than via an ABC transporter [11]. Similarly, nickel (Ni) is also taken up by a variety of pathways, including two distinct permeases and two distinct ABC importers due to its essential as a catalyst in urea metabolism [120], however, currently only one *Cyanobium* strain (NS01) contains a predicted cobalt/nickel binding protein (CK_53501).

SBPs likely associated with uptake of other microelements include a predicted SBP (CK_8059) annotated to bind either peptides or nickel, widely distributed in all picocyanobacterial strains, except for *Prochlorococcus* LLII strains. In addition, less widely distributed SBPs include a predicted molybdenum-specific protein (CK_40935) found in three *Synechococcus* strains (CC9619, KORDI-100, and WH5701), and a predicted Fe siderophore/vitamin B12-binding protein (CK_51448) found in two *Prochlorococcus* (MIT9201 and MIT9202) and one *Synechococcus* (A15-127) strains. The target substrates of all of these SBPs remain experimentally uncharacterised. Given the crucial biochemical roles of trace metals, these SBPs are highly attractive candidates for further characterisation.

## Conclusions and future research directions

### Summary

Picocyanobacteria are highly abundant primary producers found across the global ocean [4,11]. The significant role played by the picocyanobacteria in global marine primary production (>25%) cannot be understated [4,121]. Despite this, the fundamental understanding of how additional metabolic preferences (particularly for mixotrophy) complements their photoautotrophic lifestyle remains largely uncharacterised. Relative to their small genome size, picocyanobacteria dedicate large portions of their transport capacity (up to 60%) to high-affinity ABC uptake systems [17,18]. Strain-level differences observed in the genomic representation of SBPs highlight that individual strains access different portions of the nutrient pool across distinct environments. This represents a significant knowledge gap between functional predictions based on gene annotations and what occurs *in situ* in complex and dynamic marine microbial communities.

## Perspectives

- This review represents the first comprehensive use of protein structural data to define nutrient uptake functionality in picocyanobacteria. This alternative approach is particularly suitable given the low degree of sequence homology underpinning genomic annotations.
- The number and range of SBP gene clusters found in marine picocyanobacteria highlight their potential additional biochemical functionalities. Future research aimed at a systematic investigation of the predicted nutrient uptake capacity of picocyanobacteria, through structural verification of SBP substrate specificity, or the use of increasingly widespread structure prediction tools could aim to resolve ambiguity regarding their metabolic capabilities.
- Functional characterisation will further refine our understanding of how nutrient acquisition shapes ecological trajectories of picocyanobacterial communities to adapt to environmental niches, complementing ecosystem models of how these ubiquitous bacteria will respond to a changing ocean.

## Open Access Statement

Open access for this article was enabled by the participation of Macquarie University in an all-inclusive Read & Publish pilot with Portland Press and the Biochemical Society under a transformative agreement with CAUL.

## Abbreviations

ABC: ATP-binding cassette
CCM: carbon-concentrating machinery
CLOGs: clusters of orthologous genes
LL: low-light
PDB: protein data bank
SBP: substrate-binding protein
